# Supplementary figures and images for: Differential levels of IFNα subtypes in autoimmunity and viral infection
Source: Cytokine. Author manuscript; Available in PMC 2023 Aug 9. (PMC7614897; doi:10.1016/j.cyto.2021.155533)

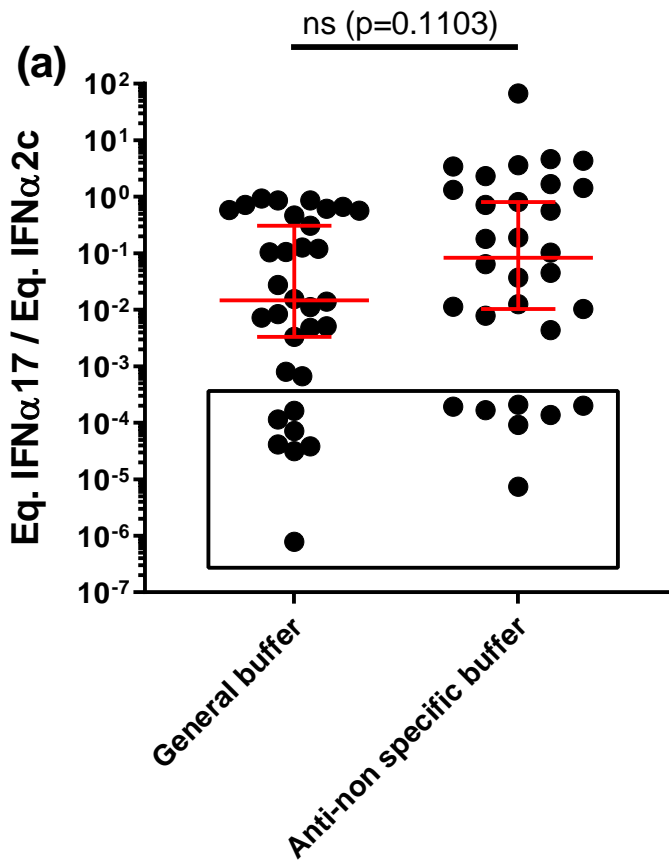

Supplement: Figure S2 [file EMS182765-supplement-Figure_S2.pdf]
