# Supplementary material for: Differential levels of IFNα subtypes in autoimmunity and viral infection
Source: Cytokine. Author manuscript; Available in PMC 2023 Aug 9. (PMC7614897; doi:10.1016/j.cyto.2021.155533)
Supplement: Supplementary Material [file EMS182765-supplement-Supplementary_Material.doc]

**Supplementary material**

**Figure S1. Abnormal IFN subtype ratios are not due to unspecific reactions**.

IFNα17/α2 protein ratios obtained using general Detector / Sample Diluent or buffer B for a selection of anti-IFNα positive and anti-IFNα negative patients.


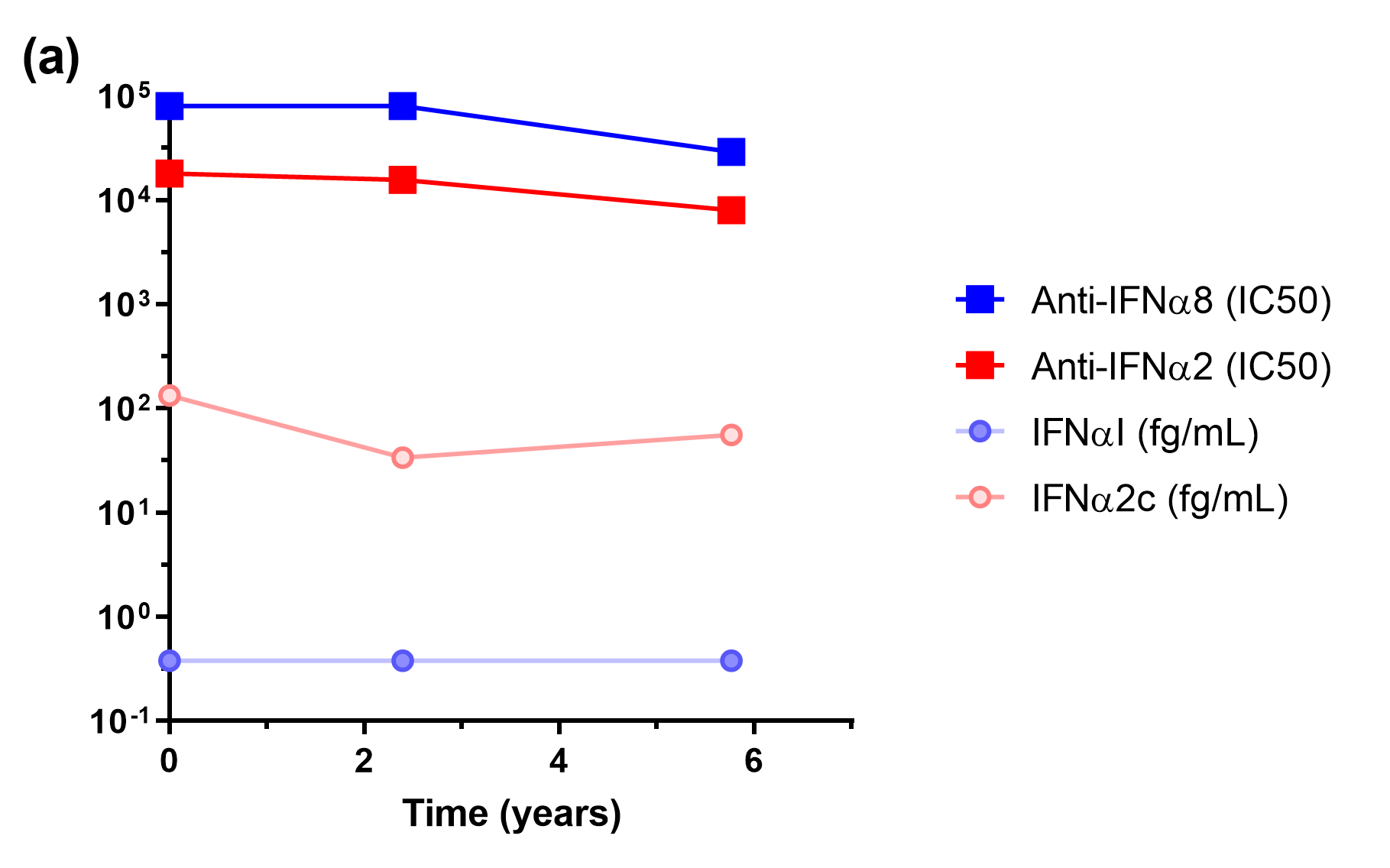
**Figure S2.** Longitudinal analysis of anti-IFNα8 and anti-IFNα2 antibody concentrations, pan-IFNα and IFNα2 assays results over time (years) in one other SLE patient.

**Table S1. Patient data sets.** For each patient included in this study, origin cohort, inclusion cohort, diagnosis, gender, age, autoantibodies presence, IFNα17 and IFNα2 concentrations, IFNα17/IFNα2 ratio, ISG score, IFN activity, and quantification results for antibodies against IFNα are done when available. LEAP (lupus extended autoimmune phenotype) cohort: see Reynolds *et al.*^16^. Rodero *et al*. cohort: see reference 13. ASSESS (assessment of systemic complications (signs) and evolution in Sjögren's syndrome) cohort: see Bost *et al.* (In review). Menon *et al*. cohort: paper in preparation. C10-08 cohort: see Sultanik *et al.^17^*. Upasani *et al*. cohort: see reference 18. UCDT: undifferentiated CTD. MCTD: mixed CTD. CTD: connective tissue disease. pSS: primary Sjögren's syndrome. SLE: systemic lupus erythematosus. JSLE: juvenile SLE. cHCV: chronic hepatitis C virus infection. CNS: central nervous system.
